# Contextual and Psychological Predictors of Militant Extremist Mindset in Youth

**DOI:** 10.3389/fpsyg.2021.622571

**Published:** 2021-02-10

**Authors:** Maša Vukčević Marković, Aleksandra Nicović, Marko Živanović

**Affiliations:** ^1^Department of Psychology, Institute of Psychology and Laboratory for the Research of Individual Differences, Faculty of Philosophy, University of Belgrade, Belgrade, Serbia; ^2^PIN—Psychosocial Innovation Network, Belgrade, Serbia

**Keywords:** radicalization, violent extremism, militant extremist mindset, loneliness, social dominance, right-wing authoritarianism, family dysfunction, hostile school environment

## Abstract

The present study aims to identify contextual and psychological factors of proneness to radicalization and violent extremism (RVE) operationalized through the Militant Extremist Mindset scale (MEM) consisting of three distinct aspects: *Proviolence* (PV), *Vile World beliefs* (VW), and *trust in Divine Power* (DP). A community sample of 271 high school students (72% females) from Belgrade and Sandžak regions in Serbia completed: (1) a 24-item MEM scale; (2) contextual measures including a 6-item scale of *family dysfunction* (FDys) and a 4-item composite measure capturing exposure to a harsh school environment and peer abuse (HSE); (3) psychological measures including the 9-item *Right-Wing Authoritarianism* scale (RWA), the 5-item *Social Dominance Orientation* scale (SDO), and the 20-item *UCLA Loneliness* scale (LON). A path analysis was conducted with contextual factors on the first and psychological factors on the second level of the model predicting the three factors of MEM. LON was positively predicted by FDys and HSE, SDO by HSE only, while RWA was positively predicted by FDys only. Contextual and psychological factors accounted for 27% of the variance in PV (LON, SDO), 15% of the variance in VW (FDys, SDO), and 31% of the variance in DP (RWA). Obtained findings reveal a complex interplay of contextual and psychological drivers in the prediction of different aspects of RVE and build upon existing knowledge on risk factors associated with RVE.

## Introduction

Radicalization refers to the process through which one adopts political, social, and religious ideation that leads to the initiation of violent acts (Demunter et al., 2019). Radical individuals become increasingly motivated to use violent means against members of an out-group or symbolic targets to achieve behavioral change and political goals (Doosje et al., 2016). Violent radicalization is defined as the process of adopting and promoting extremist beliefs for the purpose of facilitating ideology-driven violence to advance political, religious, or social change (Alcalá et al., 2017). The role of radicalization in processes leading to terrorism and its wider societal impact gained importance over the last decade (Doosje et al., 2016; Campelo et al., 2018) leading to a growing interest in the study of radicalization as a phenomenon and its drivers. The societal impact of radicalization is reflected not only in direct effects on mortality, but also in deteriorating inter- and intragroup relations, the induction of fear which can lead to a further increase of polarization between ethnic, religious, and national groups, promotion of conflict among different segments of society (Doosje et al., 2016), negative mental health outcomes (Rousseau et al., 2015; Alcalá et al., 2017), and economic losses (Frey et al., 2007).

A number of studies describe the process and propose models of radicalization as well as its underlying mechanisms (Horgan, 2008; McCauley and Moskalenko, 2008; King and Taylor, 2011; Borum, 2012; Doosje et al., 2016). Yet, there is a lack of empirical evidence supporting any specific theory, single cause, or a unifying model of radicalization. Rather, previous studies indicate that the process of radicalization stems from multiple sources and is driven by the interplay of various factors (Soliman et al., 2016; Campelo et al., 2018; Lösel et al., 2018).

Exploration of radicalization and violent extremism (RVE) was initially orientated toward the assessment of these phenomena among terrorists or members of radical groups (Klausen et al., 2016). However, the research on terrorism, despite indicating some potential pathways and models (Horgan et al., 2016), found no clear psychological profile of ‘a terrorist’ or particular trait leading to terrorism (Silke, 2010). Some authors suggested that instead of profiling one's susceptibility for RVE, the focus should be shifted toward exploring the process of radicalization and factors contributing to engaging in RVE (Horgan, 2008). In order to understand the underlying mechanisms of the process of radicalization, the additional line of research emerged, focusing on the assessment of proneness to radicalization and violent extremism in the general population and providing the evidence which suggests that ingredients of radical and violent extremism mindset are, to some extent, present in all human beings (Stankov et al., 2018). Therefore, studying potential for RVE in general population can offer new insights into this phenomena and its precursors, that could be of particular interest in post-conflict societies and regions with the history or ongoing ethnic and religious tensions (Mededović and Petrović, 2016; Stankov et al., 2019).

Youth is of particular interest in studying RVE since investigations of radical and extremist groups indicate that most newcomers are young individuals in their late teens to 30 years old (Silke, 1998). In particular, adolescents are thought to be a highly susceptible and thus vulnerable group for RVE (Campelo et al., 2018; Petrović and Stakić, 2018), due to fragile identity and self-uncertainty typical for their age (Marcia, 1980; Brown et al., 1986; Hogg et al., 2011). They are often seeking and identifying with groups that can offer strong boundaries and directive leadership which can often include radical ideology and engagement in violence (Hogg, 2014; Ellis and Ellis, 2017).

### Contextual Drivers of RVE

Literature suggests that different factors associated with the group one belongs to or relates to, such as ethnic group, peer group, or family, are linked to RVE. These factors usually include the disadvantaged position, recognition of unequal conditions and inequality, as well as relative deprivation of one's own group and exposure to group violence (Campelo et al., 2018; Lösel et al., 2018). Two of the prominent contextual drivers of RVE that can be found in the general population are unfavorable relationships within family and peers. Namely, it was demonstrated that relations with family and peers are both risk/protective factors for radicalization—those being exposed to peer violence or family dysfunction are more prone to violent tendencies and radicalization (Campelo et al., 2018). On the other hand, non-violent peer and family environments serve as a protective factor for these phenomena (Cragin et al., 2015; Lösel et al., 2018). Therefore, it could be expected that individuals who were raised in dysfunctional families and those who are exposed to a hostile social environment are more prone to embrace RVE than their peers who grew up in a more nurturing environment.

### Psychological Drivers of RVE

One of the potential psychological drivers of RVE is *right-wing authoritarianism* (RWA)—a construct which was originally described by Adorno et al. (1950) and was later refined by Altemeyer (1988, 1998). RWA is a trait-like individual-differences dimension that summarizes social attitudes that value uniformity, favor conservative, ethnocentric, and religious values, emphasize respect for authority, obedience, and authoritarian submission, promote authoritarian aggression, and often foster hostile views and attitudes toward out-groups and people who do not adhere to them (Altemeyer, 1998). Many groups that hold radical attitudes often uphold a rigid hierarchy and share conservative values. RWA itself represents a salient indicator of conservative ideology and its rigidity is best reflected in resistance to change—dogmatism, low openness to experience, needs for order, structure, and closure, as well as fear of threat (Jost et al., 2003). Thus, on a conceptual level RWA and RVE could be regarded as similar since they are both characterized by close-mindedness and rigidness, and underlie various adverse social attitudes and beliefs that are of broadly ideological nature and both oppose the fundamental values of democratic societies and universal human rights. The main difference is that RWA stands as a dimension that captures “normal” variations in ideological attitudes and beliefs, while RVE can be considered as an extreme standpoint, i.e., deviation from the norm in most of the socio-political and cultural contexts. However, some findings show that RWA and fundamentalistic tendencies share a considerable portion of variance (Altemeyer and Hunsberger, 1992) and that authoritarianism/fundamentalism emerges as one of the most prominent risk factors for radical attitudes (Wolfowicz et al., 2019). In addition to conceptual similarities between RWA and ideologies found in radical groups, empirical data shows that RWA predicts the tendency toward justification of different forms of violence, such as support for excessive use of force (Gerber and Jackson, 2017) and attitudes supporting restrictions of civil rights (Kossowska et al., 2011). Bearing in mind conceptual similarities as well as empirical findings it seems that RWA can potentially serve as “a risk factor” for engaging in RVE.

Another trait-like feature that could serve as a potential precursor of RVE is *Social dominance orientation* (SDO). SDO, defined on the basis of social dominance theory (Pratto et al., 2006) is a “general attitudinal orientation toward intergroup relations” that reflects a preference for in-group dominance and the extent to which one believes that his/her group should be superior to out-group members (Pratto et al., 1994). Similarly to RWA, SDO is a dimension underlying social or ideological attitudes and values. A conceptual link between SDO and RVE stems from the notion that their joint core features are lack of empathy, discriminatory attitudes, aggression, vindictiveness, and the readiness to support and justify the use of coercion or violence toward others, specifically out-group members (see Pratto et al., 1994, 2006; Stankov et al., 2010; Doosje et al., 2016). Therefore, attitudinal orientation or dominant values regarding intergroup relations and group dominance could be expected to be predictive of one's readiness to promote and potentially engage in aggressive acts toward out-group members (Pratto et al., 1994). SDO was shown to be the central individual differences variable that predicts acceptance or rejection of various ideologies relevant to group relations and related behaviors (Pratto et al., 1994). It was shown that SDO is related to racism and nationalism, sexism, support for military programs, opposition to women's and gay and lesbian rights (Pratto et al., 1994), intergroup violence (Henry et al., 2005), as well as bullying behavior (Goodboy et al., 2016). Moreover, findings show that SDO is not a mere effect of prejudice and discrimination against ethnic and racial outgroups, but their causal predictor (Kteily et al., 2011). Therefore, it seems that the potential of SDO in accounting for individual differences in proneness to RVE could lie in the shared values of striving for power and self-interest found in both constructs (see Stankov et al., 2010).

Finally, the literature on radicalization shows that individuals who lack social connections and are socially alienated are more likely to engage in RVE (for more see Lösel et al., 2018). In addition, subjective feelings of *loneliness* proved to be predictive of proviolence tendencies and readiness to engage in violent acts, as well as justification of the use of violence (van Tilburg et al., 2019). It could be assumed that subjective feelings of loneliness are particularly important for susceptibility to join radical groups who offer restoration of a sense of belonging, and in return, ask for the promotion, justification, and use of violence by their members (Doosje et al., 2016). In other words, young individuals who feel lonely, isolated, excluded from an ingroup, detached and estranged from society can be particularly susceptible to RVE. They are prone to seek solace in various radical ideologies and groups which promise to restore purpose to their followers (Kruglanski et al., 2014) and those who comply with their ideological imperatives. On the other side, more social connections and identification with peers proved to be protective for radicalization and violent acts (Williams et al., 2015; Knight et al., 2017; Lösel et al., 2018).

### Current Study

Despite a growing body of evidence on psychological and contextual drivers of RVE, there is a lack of integration of these findings by examining their complex interplay in addition to their separate contribution as risk factors for radicalization. Only by looking at both psychological and contextual drivers of RVE as well as their interplay we could better understand the social and psychological “breeding ground” and cascade of experiences that could trigger vulnerable individuals to come closer to radical groups, embrace radical attitudes and viewpoints, and potentially engage in RVE.

Therefore, in this study, we aim to propose and test the unified prediction model of drivers of RVE, built upon existing theoretical models and a body of empirical evidence, including two broad clusters of risk factors—contextual and psychological risk factors, while taking into account their interplay. The first group of risk factors, labeled as contextual risk factors, included family dysfunction, a hostile school environment, and peer abuse. These factors were proven to be relevant not just for RVE, but also for various psychological outcomes which are shown to be related to these phenomena. The second group of risk factors labeled psychological risk factors, included right-wing authoritarianism and social dominance orientation, as well as feelings of social isolation and loneliness which were found to be predictive of RVE in previous studies.

Despite conceptual similarities and the body of evidence showing the relevance of ideology-related psychological factors for a wide range of social outcomes, the evidence on the role of SDO and RWA in the prediction of different aspects of radicalization is lacking. Here we hypothesized that SDO and RWA coupled with feelings of social estrangement and lack of a sense of belonging make one especially susceptible to ideas typically found in followers of radical and violent groups. Relying on the large body of evidence showing that unfavorable relationships within the family and peer groups which, in addition to being risk factors for radicalization (Campelo et al., 2018), also affect one's well-being by causing various emotional, mental health, behavioral and interpersonal problems (Segrin et al., 2012; Wang et al., 2020), we hypothesized that unfavorable relations within family and peer group will also make one susceptible to radicalization and violent extremism. In the present study, we tested a complex interplay of contextual and psychological factors in the prediction of RVE. Namely, since previous studies rarely explored the interrelation between these two groups of drivers of radicalization, thus failing to offer a comprehensive empirical approach to underlying mechanisms leading to RVE, we aimed to bridge the gap in knowledge on their interplay. Specifically, we hypothesized that broad contextual factors, in addition to directly making one more prone to embrace RVE, can serve as precursors of RWA and SDO as well as feelings of loneliness and estrangement which could further nudge a person toward radicalization. Therefore, here we tested the assumption that contextual factors at least partially shape individual thinking patterns (Moghaddam, 2005; Borum, 2012) that could lead to RVE. For instance, an unfavorable family environment during childhood was shown to predict loneliness in adulthood (Segrin et al., 2012) and was found to be related to depression and other mental health problems (Wang et al., 2020). In addition, there is evidence that young people who come from violent families tend to see the world and other people as hostile and untrustworthy (Steinberg, 2000). Furthermore, studies have shown that many young people who express antisocial and violent behavior come from families with a history of violence, abuse, or some other form of family dysfunction (Steinberg, 2000; Okour and Hijazi, 2009; Sousa et al., 2010). In addition to being directly related to radicalization among youth, it has been shown that relationship with peers is a significant predictor of social isolation (Lösel et al., 2018) and that young people who have been exposed to peer violence later suffer from a greater feeling of loneliness (Estévez et al., 2009).

Since there is no unique approach to the measurement of radicalization and violent extremism, we chose to rely on the psychological concept of Militant Extremist Mindset (MEM, Stankov et al., 2010) as an output measure as it relatively comprehensively captures the mindset of those prone to RVE. Stankov et al. (2010) describe MEM as a pattern of beliefs, feelings, thoughts, and motivations that can be aroused under certain conditions and lead to violent behavior. The MEM is thought to capture individual differences in acceptance of radical and extremist ideology as well as the extent of radicalization in the general population (Stankov et al., 2018). It captures three dimensions of radicalization: (1) *Proviolence* reflects justification, acceptance, and advocacy for the use of violence in the context of revenge or redemption; (2) *Vile World* captures the belief that something is seriously wrong with the world in which we live, that today's world is miserable and evil, and (3) *Divine Power* summarizes strict moral principles and belief in divine power, God, and paradise as a justification for the use of violence. The authors of MEM argue that taking all three factors into account is necessary to understand radicalization. Justification and advocacy for violence in the case of a strong belief that the world is an unjust and evil place followed by feelings of threat and danger are thought to lead to a higher probability of engaging in violence while seeking justification for such actions (Stankov et al., 2018).

## Method

### Participants and Procedure

The sample consisted of 271 high school students (72% females) between 15 and 18 years of age (*M* = 16.30, *SD* = 0.69) from the areas of Belgrade and Sandžak in Serbia. Participants were recruited in coordination with school psychologists and approached at a predefined time during school hours. All participants were invited to take part in the study voluntarily. Students, as well as their teachers, parents, and legal guardians were informed about the purpose of the study, and their parents or legal guardians gave their informed consent. Participants completed a battery of questionnaires during group sessions on school premises. Data collection was conducted by trained psychologists, and after completing the questionnaires all participants were debriefed. All personal information was kept confidential and all the data were anonymized prior to analyses. All procedures adhered to the Declaration of Helsinki standards and the study was approved by the Institutional Review Board of the Department of Psychology, University of Belgrade, Serbia (Protocol #2019-037).

### Measures

#### Contextual Predictors

*Family dysfunction* was assessed using a 6-item (e.g., “*Physical and verbal conflicts happened often in my home while I was growing up”*) subscale from a more comprehensive Bad socialization scale, which has been validated and widely used in the local context (see Kneževic, 2003; Mededović, 2019). The family dysfunction subscale measures various dysfunctional parental practices during the participant's childhood such as parental neglect and parental maltreatment. Each item was rated on a 5-point Likert scale from 1—completely false to 5—completely true, where high scores indicated higher family dysfunction.

*The harsh school environment* (HSE) was assessed using four items. Two items of the Bully/Victim questionnaire (Olweus, 1996) were used (“*How often have you been verbally bullied by someone at school in the past school year”* and “*How often have you been physically bullied by someone at school in the past school year”*), as well as an additional item capturing the frequency of suffering verbal and physical abuse and fights in one's class (“*How often there are physical and verbal conflicts in your class?”*). Items were rated on a 5—point Likert scale (*1*—*never, 2*—*once or twice, 3*—*two to three times a month, 4*—*approximately once a week, and 5*—*few times a week*). In addition, a single-item measure of the general feeling of safety in the school environment was added to the scale (“*Do you generally feel safe at school?”*) rated on a 5—point Likert scale (*1*—*I never feel safe at school to 5*—*I always feel safe at school*). A composite score of these four items was used as a measure of HSE, where higher values indicated a more violent and harsh school environment.

#### Psychological Predictors

*Right-wing authoritarianism* was measured using a 9-item short version of the RWA scale (e.g., “*The most important values that children have to learn are obedience and respect for authority”*) assessing the tendency to respect and obey authority and support conservative values (Altemeyer, 1988; Todosijević, 2013). Each statement was rated using a 5-point scale ranging from *1*—*completely false to 5*—*completely true*, and higher scores indicated greater acceptance of RWA attitudes. The abundance of empirical findings has shown that the scale is valid and psychometrically sound (e.g., Altemeyer, 1988; Todosijević, 2013).

*Social Dominance Orientation (SDO)* was measured using 5 items of the Group Dominance subscale (e.g., “*Some groups of people are just less worthy than others”*), that conceptualizes it as the generalized attitude toward intergroup relations, accepting or opposing hierarchies as a natural world order (Pratto et al., 1994; Todosijević, 2013). Each item was rated on a 5-point Likert scale from *1*—*completely false to 5*—*completely true*, where high scores indicated attitudes supporting group inequality. The validity of the scale is very well-documented in the literature (e.g., Pratto et al., 1994; Henry et al., 2005), as well as in local context (e.g., Todosijević, 2013).

*Loneliness* was assessed using the UCLA Loneliness Scale that operationalizes subjective feelings of loneliness and feelings of social isolation (Russell et al., 1980; Turner et al., 2010). It consists of 20 items (e.g., “*I lack companionship”*), accompanied by a 4-point Likert scale ranging from *1*—*never* to *4*—*often*. Higher scores on this scale indicated a greater feeling of social isolation and loneliness. A number of studies proved that the scale is both psychometrically sound and valid, with high test-retest reliability that indicate it rather captures prolonged feelings of loneliness than just the current emotional state (Russell et al., 1980; Russell, 1996).

#### Outcome Measure

*Militant Extremist Mindset*—*MEM* was assessed using the revised MEM scale (Stankov et al., 2010, 2018), which consists of 24 items designed to measure beliefs typical of the militant extremist thinking pattern. MEM items are grouped into three subscales: (1) *Proviolence* (10 items) which measures readiness to use violence in order to solve social problems (e.g., “*Armed struggle is the only way that youths can redeem themselves and their society”*); (2) *Divine Power* (8 items) which assesses the justification of unfriendly thoughts and violent acts in order to resort to something sacred by calling upon God and higher moral principles (e.g., “*At a critical moment, divine power will step in to help our people”*), and (3) *Vile World* (6 items) which evaluates the degree to which one believes that the present-day world is vile and reflects a grudge which provides justification for anger (e.g., “*The present-day world is vile and miserable”*). Each statement is rated on a 5-point Likert scale ranging from *1*—*strongly disagree*, to *5*—*strongly agree*. Three separate scores for each of the corresponding MEM factors were calculated and higher scores indicated greater proviolent tendencies/belief in vile world/ belief in divine power. The validity of the construct was demonstrated on a wide range of relevant socio-psychological constructs (see Stankov et al., 2010).

## Results

The descriptive statistics and zero-order correlations of three factors of MEM and contextual and psychological measures used in this study are presented in Table 1. Results showed that young males scored significantly higher than young females on the proviolence subscale [*t*_(269)_ =5.34, *p* < 0.001], while between-group differences were not observed for vile world beliefs [*t*_(269)_ =0.13, *p* = 0.90], and divine power [*t*_(269)_ =1.00, *p* = 0.32]. All contextual and psychological variables, except RWA, demonstrated positive asymmetry of the distribution of scores indicating that the majority of participants were raised in functional families and were not exposed to peer violence on a daily basis. Similarly, the majority of participants exhibited low scores for loneliness as well as for SDO. On the other hand, the majority was grouped in the higher score-range for the vile world and divine power domains, while scores for proviolence grouped in the lower score-range. Due to high values of skewness and kurtosis, all variables were normalized using the Rankit formula. Cronbach alpha coefficients for each measure used are given on the main diagonal of Table 1. Bearing in mind, the number of items per scale, all measures demonstrated acceptable internal consistencies, ranging from 0.54 for HSE to 0.85 for the vile world.

**Table 1:** Descriptive statistics for measures used (*N* = 271).

**Measures**	***M***	***SD***	***Sk***	***Ku***	**FDys**	**HSE**	**SDO**	**RWA**	**LON**	**PV**	**VW**	**DP**
FDys	1.59	0.68	1.80	4.26	*0.68*	0.36^**^	0.16^*^	0.11	0.39^**^	0.22^**^	0.33^**^	0.03
HSE	1.70	0.66	1.37	1.64		*0.54*	0.23^**^	−0.06	0.34^**^	0.27^**^	0.21^**^	−0.09
SDO	2.17	0.88	0.75	0.26			*0.63*	0.26^**^	0.11	0.45^**^	0.24^**^	0.03
RWA	3.05	0.81	−0.16	−0.33				*0.78*	0.03	0.10	0.06	0.55^**^
LON	2.30	0.42	0.94	1.02					*0.80*	0.28^**^	0.10	−0.03
PV	1.71	0.68	1.93	5.01						*0.80*	0.23^**^	−0.14^*^
VW	3.46	0.94	−0.50	−0.23							*0.85*	−0.01
DP	2.99	0.81	−0.42	−0.33								*0.77*

Diagonal values—Cronbach alpha;

* p < 0.05;

** *p < 0.01*.

Low to moderate zero-order Pearson correlations between normalized scores of contextual and psychological factors were observed. Namely, family dysfunction was positively correlated with HSE, SDO, and loneliness, while a trend-level correlation was obtained for RWA (*p* = 0.07). A similar pattern of correlations was observed for HSE. The correlations between divine power and right-wing authoritarianism, as well as between proviolence and social dominance orientation scores were moderate to high. Somewhat lower positive correlations were obtained for the proviolence and vile world with both measures of contextual factors. Similarly, a low to moderate positive correlation was obtained between the vile world subscale and SDO, as well as between proviolence and loneliness. Contextual variables and SDO, but not RWA achieved low to moderate positive correlations with proviolence and vile world beliefs, while loneliness was correlated with proviolence only. Finally, RWA proved to be significantly correlated with divine power, but not the other two dimensions of MEM.

**Figure 1:**
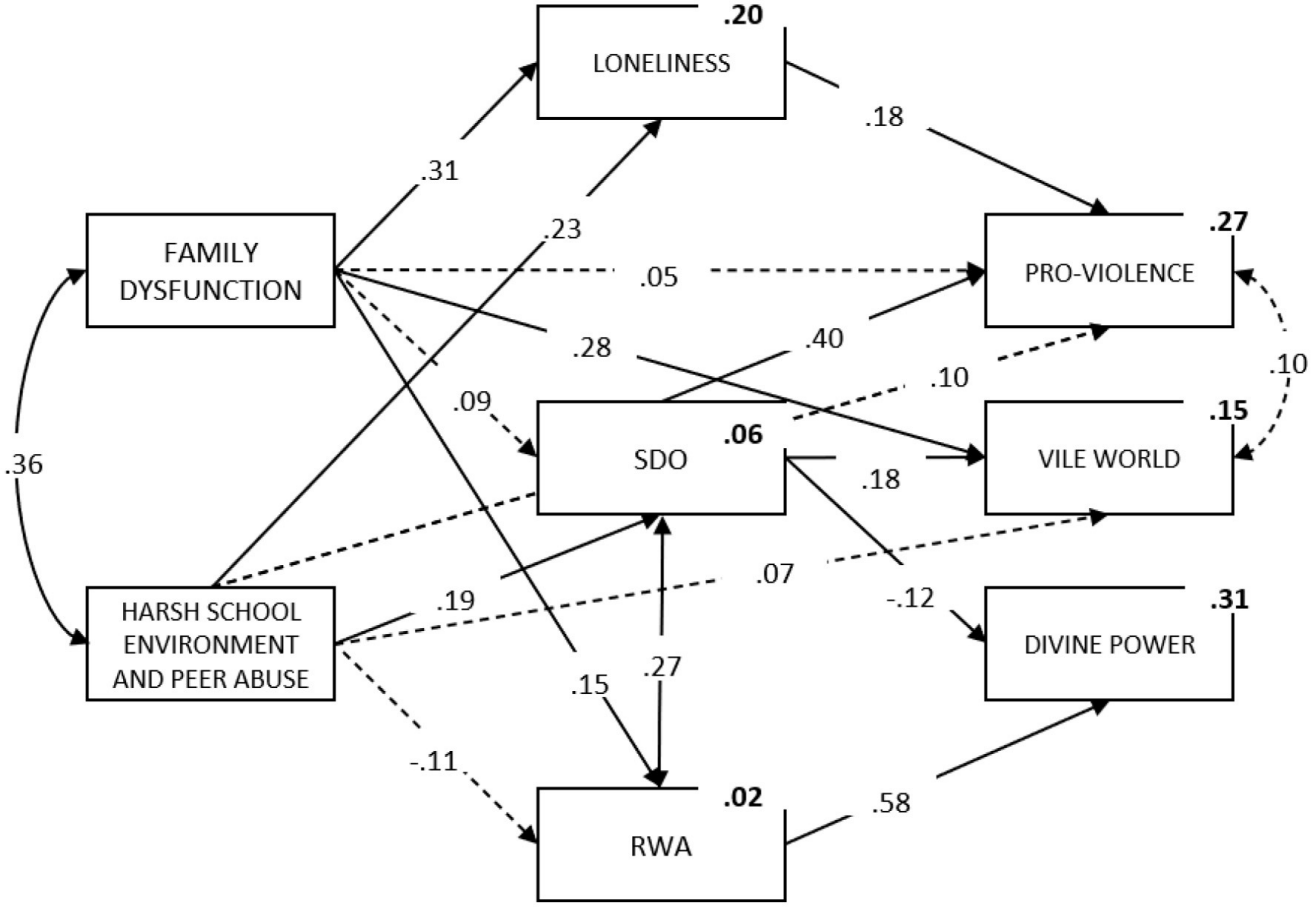
Prediction of MEM. Statistically significant coefficients are marked by full arrows; Squared multiple correlations are shown in *bold*.

The prediction model of MEM was conceptualized as a three-level model (Figure 1). The first, contextual level consisted of past—*family dysfunction*, and current—*hostile school environment and peer abuse*, adverse contextual effects. The second, psychological level, consisted of ideological factors, specifically *right-wing authoritarianism* and *social dominance orientation*, as well as *loneliness* as a marker of current social deprivation. Finally, on the third level of the model three domains of MEM were specified as outcome variables. For the contextual factors, both direct and indirect effects on MEM were assumed. On the other hand, for psychological factors, only direct effects on MEM factors were specified. In other words, based on previous findings showing that unfavorable family and peer environments have an impact on RVE, it was assumed that the level of aversive past and current experiences can directly shape proviolent tendencies and vile world beliefs. In addition, these contextual variables are thought to indirectly affect different aspects of MEM through their impact on feelings of loneliness and estrangement, proneness to social domination, and rigid value structure summarized in right-wing ideologies. These psychological aspects are hypothesized to further predispose one to militant and extremist beliefs. More specifically, it was assumed that loneliness directly promotes proviolent tendencies, SDO affects all three aspects of MEM, and RWA has an effect on divine power.

To test this model, a Path analysis, using IBM SPSS Amos 21.0 software was conducted. Several fit indices of model fit were consulted, namely, Chi-square statistic, Comparative Fit Index (*CFI*), Tucker-Lewis Fit Index (*TLI*), Root Mean Square Error of Approximation (*RMSEA*), and Standardized Root Mean Square Residual (*SRMR*). We evaluated the specified model using the following criteria—non-significant Chi-square, *CFI*, and *TLI* ≥ 0.95, *RMSEA* < 0.06, and *SRMR* < 0.08 (Hu and Bentler, 1999).

The results showed that the model had a very good fit as suggested by both relative and absolute indices [$\chi_{\left(10\right)}^{2}$ = 11.38, *p* = 0.33, *CFI* = 0.99, *TLI* = 0.99, *RMSEA* = 0.02, CI 90%: 0.00–0.07, *SRMR* = 0.03].

The model shows that contextual factors exhibited significant relationships with psychological domains. Among psychological variables, loneliness proved to be best predicted by contextual factors. Namely, it was significantly predicted by both family dysfunction and harsh school environment. In addition, family dysfunction predicted RWA, while harsh school environment significantly predicted SDO. Finally, family dysfunction predicted vile world views, while a harsh school environment demonstrated only a trend-level effect on proviolent tendencies (*p* = 0.09).

Both groups of predictors taken together (Figure 1) accounted for 26.6% of the variance of proviolent tendencies. More specifically, significant effects of SDO and loneliness on proviolence were observed. The effect of family dysfunction was fully mediated by loneliness, while the effect of the harsh school environment was only partially mediated by psychological variables (indirect effect of 0.12). Contextual and psychological factors accounted for 14.9% of the variance of vile world beliefs—SDO and family dysfunction emerged as significant predictors, however family dysfunction had a stronger direct effect. Finally, SDO and RWA accounted for 31.1% of the variance in divine power beliefs, with SDO acting as a suppressor variable and RWA being a strong positive predictor of divine power.

## Discussion

Even though previous studies have shown that radicalization is a complex phenomenon that is multi-determined and driven by multiple factors (Soliman et al., 2016; Campelo et al., 2018; Lösel et al., 2018), so far little is known about the relative contribution of individual risk factors to the prediction of RVE, and their interplay in paving the way to violent behaviors and extremist beliefs. Therefore, this study aimed to shed light on prominent contextual and psychological factors that can be predictive of RVE based on previous studies. Specifically, the present study proposed and evaluated the unified model of drivers of radicalization, including two groups of risk factors—those related to one's immediate social environment summarizing past and current adverse experiences in the family and school context, i.e., contextual factors; and ideology-related risk-factors that were hypothesized to reflect core beliefs typically found in radical ideologies such as right-wing authoritarianism and social dominance orientation. In addition to these ideological drivers, we examined the relative contribution of feelings of social estrangement and loneliness in the prediction of RVE as previous studies pointed out that young individuals who lack a sense of belonging and feel socially isolated are more prone to embrace the values of radical groups and ideologies (Doosje et al., 2016; Bélanger et al., 2019).

The results point to the importance of contextual factors in both the prediction of psychological drivers of RVE and different aspects of MEM. Namely, it has been shown that the roots of loneliness can, at least in part, be found in aversive family experiences and unfavorable interactions with peers reflected in peer abuse and a harsh school environment. This finding is in keeping up with the body of evidence linking family dysfunction and a harsh school environment to loneliness (Estévez et al., 2009; Lösel et al., 2018; Wang et al., 2020). It can be assumed that underlying mechanisms of the effects parents and family can have on their offspring's loneliness are both direct, through genetic transmission, and indirect, through the creation of dysfunctional family environments in which patterns of communication are more restrictive and less effective (Segrin et al., 2012). Less supportive and functional environments, in which people do not feel free to express themselves, cause feelings of alienation and a lack of understanding, which are crucial components of loneliness (Segrin et al., 2012; Wang et al., 2020).

On the other hand, the ideological belief system typically associated with social dominance orientation and right-wing authoritarianism proved to be far less rooted in current and past hostility experienced at home or in the school context. This finding should not come as a surprise since SDO and RWA are thought to be of dispositional nature, and thus are relatively strongly rooted in other endogenous personality structures (Heaven and Bucci, 2001; Duckitt et al., 2002; Jost et al., 2003; Ekehammar et al., 2004; Duriez and Soenens, 2006). Their positive relationship, obtained here, validates the work of Altemeyer (2004) who pointed out numerous links between authoritarians and dominants, including sharing racial, ethnic, sexist, and sexual orientation prejudices, conservative economic philosophies, and preference of the right-wing political parties (Altemeyer, 2004). We found that individuals who are raised in dysfunctional families are somewhat more prone to embrace right-wing authoritarianism, probably because they are seeking a clear and well-ordered social structure and clear-cut values and beliefs that can provide them safety and certainty—capacities they were probably denied or misinterpreted within their families (Duriez et al., 2007). On the other hand, individuals who are frequently exposed to a hostile school environment proved to be more inclined to attitudes reflected in social dominance orientation. Although relatively weakly correlated, it can be assumed that the constant frustration caused by lack of safety and suffering humiliation due to frequent peer violence in the school context can emphasize a system of beliefs which reflects a desire for power and domination, and which favors anti-egalitarianism, hierarchy, and domination as a way to combat unfavorable social circumstances.

Apart from being predictive of psychological variables, family dysfunction proved to be the main determinant of seeing the world as an evil, unjust, and miserable place. The relationship between family dysfunction and vile world beliefs should be understood in the context of the nature of this dimension. Namely, Stankov (2018) discussed the tendency to perceive the world as evil as a potential consequence of psychological upheavals during adolescence. It is thus very straightforward why aversive family experiences and prolonged deleterious interactions with parents can, besides negatively affecting self-concept and feelings about themselves (Wang et al., 2020) and interpersonal relations (Miller et al., 2000), negatively affect concepts of the world and translate and generalize into view on the world as an evil and unfair place—a perception which could further serve as a rationalization of one's own violent and immoral behaviors (Saucier et al., 2009).

Loneliness and social dominance orientation emerged as the most prominent predictors of proviolence tendencies. Namely, the results showed that the individuals who feel socially isolated and estranged from society are the ones who show a greater predisposition for justification and use of violence. The relationship between loneliness and proviolent tendencies may be particularly important as it could reflect one of the mechanisms that radical groups use when recruiting new members—promising them a sense of belonging and offering them respect and status (Doosje et al., 2016). Therefore, loneliness can be understood as an important factor that drives the quest for belonging, purpose, and meaning, which can be exploited by certain groups who push vulnerable individuals toward radical worldviews and use them to achieve their goals by violent means (Kruglanski et al., 2014). Studies showing that more social connections and identification with peers are protective for radicalization and violent acts (Williams et al., 2015; Knight et al., 2017; Lösel et al., 2018) provide additional evidence supporting this perspective.

Social dominance orientation was the only significant predictor of both proviolence tendencies and vile world beliefs while serving as a suppressor variable in the prediction of divine power. Its predictive value for antisocial attitudes and beliefs is consistent with previous findings showing that SDO is related to less concern for others, nationalism, sexism, support for military programs (Pratto et al., 1994), racism (Duriez and Soenens, 2009) intergroup violence (Henry et al., 2005), excessive use of force (Gerber and Jackson, 2017), and bullying behavior (Goodboy et al., 2016). Here social dominance orientation, on one side, coupled with loneliness was predictive of proviolent tendencies, and on the other, coupled with family dysfunction predicted vile world beliefs. We believe that, in this context, the relationship between SDO and proviolence attitudes, on one hand, and with vile world beliefs on the other, should be understood as a projection of aggressive impulses arising from the dissatisfaction and chronic frustration associated with disadvantaged social circumstances. Namely, as the results demonstrated, individuals coming from disadvantaged social backgrounds are more prone to feel isolated/lonely and are more predisposed to develop resentment and feelings of injustice, as well as to hold a grudge that provides justification for anger toward certain groups. In order to cope with and control these negative feelings, they seem to resort to the simplest and quickest fix at hand in this vile world as they see it by simply shifting the roles, i.e., shifting themselves from the socially disadvantaged to the dominant and socially advantaged. Therefore, it seems that the common core of social dominance orientation and proviolence can be found in the aggressive ambition for power and self-interest (see Stankov et al., 2010).

Finally, belief in divine power proved to be very well-predicted by right-wing authoritarianism. Bearing in mind conceptual similarities between divine power as a dimension of MEM and religiosity (Stankov et al., 2010), these results are in line with the evidence showing positive relationship between RWA and religiosity (Heaven et al., 2011; Harnish et al., 2018) since both represent hallmarks of conservative syndrome (Stankov, 2009, 2018). So it seems that conservative values that underlie both RWA and religiosity can serve as a justification of violent acts and unfriendly thoughts by calling upon higher force and moral principles in resorting to something sacred but not necessarily as drivers of violence *per se*, which seems to be mainly fueled by aspiration for power and domination.

Several limitations of the present study should be noted. Firstly, both contextual measures, all three domains of MEM, and SDO demonstrated non-normality and restriction of variance in our sample thus affecting the magnitude of correlation coefficients obtained in all analyses. This issue was especially prominent for the measure of family dysfunction since the vast majority of participants reported being raised in more or less well-functioning families. Based on the relatively high restriction of variance in this measure, we can assume that the effects of family dysfunction on both psychological drivers of RVE as well as domains of MEM could only be higher than the ones reported here. In addition, the gender disproportion in our sample greatly limits the generalizability of obtained results especially bearing in mind that our, as well as previous findings, have shown that men are more prone to proviolence than women (Stankov et al., 2010). To overcome these limitations and increase the generalizability of findings, future studies should cross-validate the proposed model of prediction of RVE on larger, more diverse, and more gender-balanced samples. Next, one could argue that the results of the current study are limited by the socio-political/cultural context in which the study was conducted. However, we believe that the generalizability of obtained results is an open empirical question which needs to be addressed in future studies. Furthermore, one can argue that using retrospective measures of family dysfunction does not provide reliable and valid data on aversive past experiences and may make obtained results biased toward present family interactions and/or present psychological states. Still, we believe that family dysfunction is a “trait-like” feature of a family since, by definition, dysfunctional families are stable in the typical behavioral and emotional patterns of their members. However, future studies should aim to explore the relationship using more objective and focal indicators of family dysfunction obtained from different sources. Finally, since we were primarily focused on contextual, ideological and interaction-related predictors of RVE, future studies should include some of the important personality variables (e.g., Big Five) that were not covered by this study since previous findings have shown that they are predictive of ideological traits as well (Heaven and Bucci, 2001; Ekehammar et al., 2004; Duriez and Soenens, 2006; Teppers et al., 2013) and could potentially be of incremental value for the prediction of radicalization and violent extremism (see Stankov et al., 2010).

## Conclusion

The present study provides important empirical evidence on risk factors associated with RVE and their interplay and confirms the role of both contextual and psychological factors in predisposing vulnerable individuals from the general population to radicalization and violent extremism. Obtained results highlight the importance of linking RVE to social context, i.e., current and past aversive interpersonal experiences as well as existential experiences of loneliness and identification with ideologies usually found in militant and radical groups. Understanding the pathways in which various contextual and psychological factors and their interplay shape readiness for engagement in RVE, or act as protective factors against RVE, is of the utmost importance for informing future data-driven programs countering radicalization and violent extremism, which are, at this stage of research, underdeveloped and underrepresented in current interventions models (van Tilburg et al., 2019).

## Data Availability Statement

The raw data supporting the conclusions of this article will be made available by the authors, without undue reservation.

## Ethics Statement

The studies involving human participants were reviewed and approved by Institutional Review Board of the Department of Psychology, University of Belgrade, Serbia (Protocol #2019-037). Written informed consent to participate in this study was provided by the participants' legal guardian/next of kin.

## Author Contributions

MV and AN contributed equally to the conception and the design of work, drafted the article, while MŽ did the data analysis and critically reviewed and revised the article. All authors read and approved the final version submitted for publishing.

## Conflict of Interest

The authors declare that the research was conducted in the absence of any commercial or financial relationships that could be construed as a potential conflict of interest.
